# Management of vesicoenteric fistulas arising from perforated Meckel’s diverticulum: a report of a case and review of the literature

**DOI:** 10.1093/omcr/omad155

**Published:** 2024-02-16

**Authors:** Dimitrios Diamantidis, Nikolaos Papatheodorou, Panagiotis Kostoglou, Georgios Tsakaldimis, Sotirios Botaitis

**Affiliations:** Department of Urology, University Hospital of Alexandroupolis, Democritus University of Thrace, Alexandroupolis, Greece; 1st General Surgery Department, University Hospital of Alexandroupolis, Democritus University of Thrace, Alexandroupolis, Greece; Department of Vascular Surgery, University Hospital Erlangen, Friedrich-Alexander-Universität Erlangen-Nürnberg (FAU), Erlangen, Germany; 1st General Surgery Department, University Hospital of Alexandroupolis, Democritus University of Thrace, Alexandroupolis, Greece; Department of Urology, University Hospital of Alexandroupolis, Democritus University of Thrace, Alexandroupolis, Greece; 1st General Surgery Department, University Hospital of Alexandroupolis, Democritus University of Thrace, Alexandroupolis, Greece

## Abstract

Vesicoenteric fistulas are rare, with an incidence of 0.1%–0.2% in the general population, and Meckel’s diverticulum is a rare cause, accounting for less than 5% of cases with challenging diagnosis due to atypical symptoms at the admission. This article presents a case of a vesicoenteric fistula formation between Meckel’s diverticulum perforated by a foreign body and urinary bladder in a 38-years-old Caucasian male admitted to emergency department due to colicky abdominal pain located in the lower abdomen. An extensive review of the literature was conducted referring all the cases of vesicoenteric fistula incorporating Meckel’s diverticulum to elucidate the clinical characteristics, explore the diagnostic yield, and to summarize the therapeutic approach.

## INTRODUCTION

Meckel’s diverticulum is a rare entity that affects the gastrointestinal tract and characterized by a blind pouch protruding from the wall of the small intestine approximately two inches long. It is usually caused by failure of the omphalomesenteric duct to obliterate, and usually being found within two feet from ileocecal valve. Although it is usually asymptomatic, it can lead to complications such as ulceration, bleeding, and even vesicoenteric fistula formation. Vesicoenteric fistulas are abnormal connections between the urinary bladder and an intestinal (small bowel) segment that could cause the leakage of urine into the intestine [1–4]. They pose a scarce pathology throughout the literature, with an incidence rate of 0.1%–0.2% in general population. They develop between Meckel’s diverticulum and urinary bladder, in less than 5% of cases [2]. Symptoms of vesicoenteric fistulas can include abdominal pain, fever, urinary retention, and vomiting [5]. These conditions can be diagnosed further through a combination of clinical examination, laboratory, and imaging tests. Treatment of vesicoenteric fistulas due to Meckel’s diverticulum typically involves the resection of the affected intestinal portion and repair of the fistula [6]. In some cases, laparoscopic surgery can be used successfully to treat these fistulas [5, 7]. This article reports a rare case of a male patient suffering from an unknown until the point of the admission vesicoenteric fistula developed between the perforated by foreign body Meckel’s diverticulum and urinary bladder. Additionally, an extensive review of the literature using PubMed library was conducted using the keywords ‘enterovesical AND fistula AND meckel AND diverticulum’, ‘foreign AND body AND fistula AND meckel AND diverticulum AND vesicoenteric OR enterovesical’, ‘foreign AND body AND fistula AND meckel AND diverticulum’ and ‘vesicoenteric AND fistula AND meckel AND diverticulum’ in the title and abstract on June 2023. Moreover, the reference lists of the eligible studies and relevant review articles were cross-checked to identify all prior reported case reports of vesicoenteric fistula from Meckel diverticulum, and to determine the set of symptoms, the different diagnostic tools, and the surgical approach.

## CASE PRESENTATION

**Figure 1:**
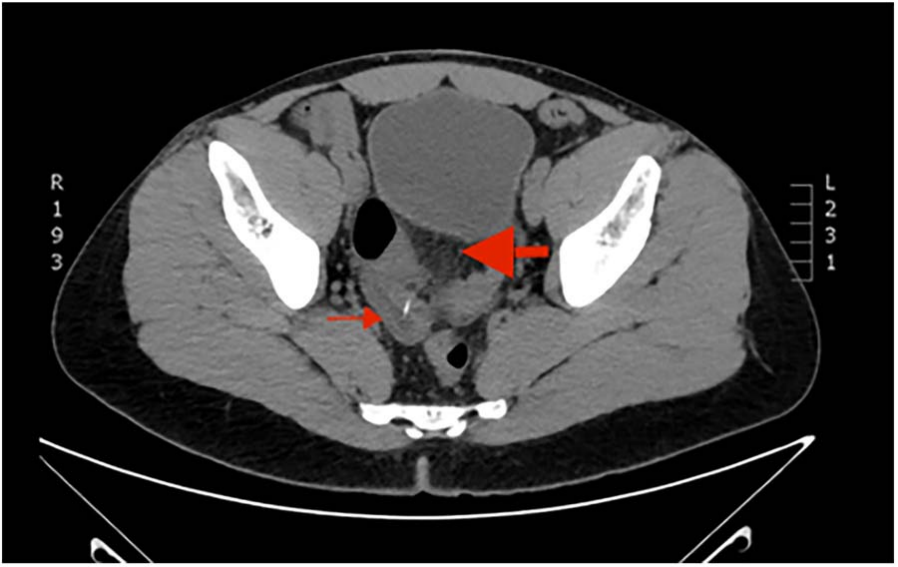
Computed Tomography of the abdomen: a blind intestinal loop (thin arrow) was attributed to Meckel’s diverticulum. Concomitant focal thickening as well as edema of the intestinal wall and the surrounding mesenterial fat (thick arrow).

**Figure 2:**
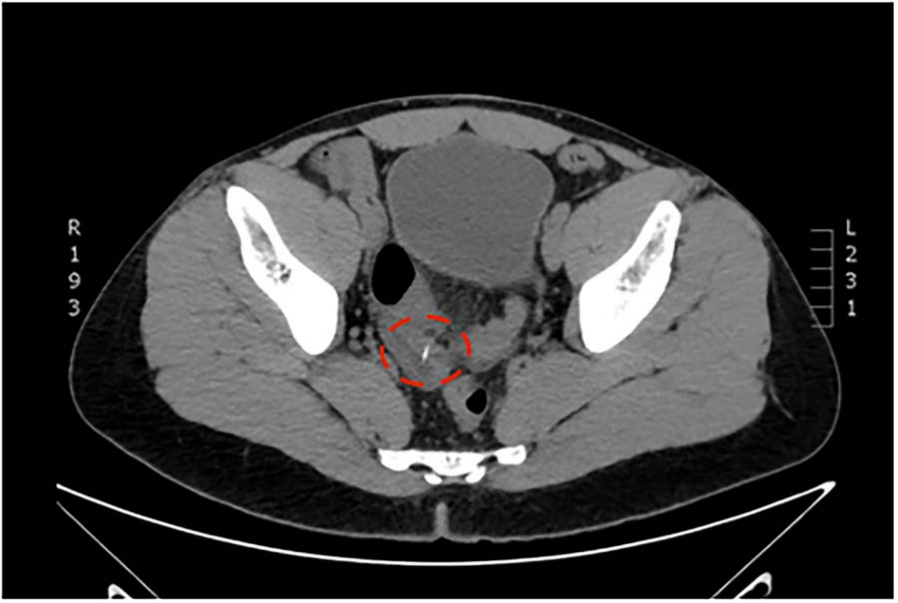
Computed Tomography of the abdomen: a thin elongated hyperdense nodule about 2 cm long perforating the intestinal wall, attributed to a foreign body (fish-bone).

A 38-year-old Caucasian male patient was admitted to the emergency department of the University Hospital of Alexandroupolis with colicky abdominal pain, ongoing for three weeks. The patient reported slight remission of the symptoms the last two weeks and exacerbation of them two days before his admission. No active bleeding, history of hematochezia, faecaluria or pneumaturia was reported by the patient. The patient was afebrile, without accompanying nausea or vomiting. Vital parameters were recorded: blood pressure measurement 125/80 mm Hg, oxygen saturation rate 97% and pulse rate of 87 beats per minute. During the clinical examination, the abdomen was distended and tympanic, with intense tenderness in the hypogastrium and right and left iliac fossa. Positive McBurney and Rovsing signs were found, as well as decreased bowel sounds during auscultation. The laboratory tests results revealed leukocytosis, with a polymorphonuclear type, and increased inflammation indices. Table 1 summarizes the results of the laboratory tests. The emergent abdominal computed tomography (CT) revealed a thin elongated radiopaque formation about 2 cm long, that could be a foreign body, protruding from a blind small bowel loop (attributed to Meckel’s diverticulum, Fig. 1) into the pelvic cavity (Fig. 2). Focal thickening as well as edema of the intestinal wall and the surrounding mesenterial fat was revealed, without any extraluminal air bubbles or intra-abdominal fluid collections (Fig. 1).

**Table 1:** Laboratory test results

		Normal values
White blood count	19.29 K/μl	3.5–10.8 K/μl
Neutrophils	84.6%	40%–75%
Serum glucose	88 mg/dl	70–100 mg/dl
Urea	23 mg/dl	20–50 mg/dl
Creatinine	0.8 mg/dl	0.8–1.4 mg/dl
C-reactive protein	3.06 mg/dl	<1 mg/dl
Aspartate aminotransferase	22 U/l	<40 U/l
Alanine aminotransferase	20 U/l	<35 U/l
Na^+^	139 mmol/l	136–146 mmol/l
K^+^	4.2 mmol/l	3.5–5.2 mmol/l
Lactate	3.8 mmol/l	<1.7 mmol/l

**Figure 3:**
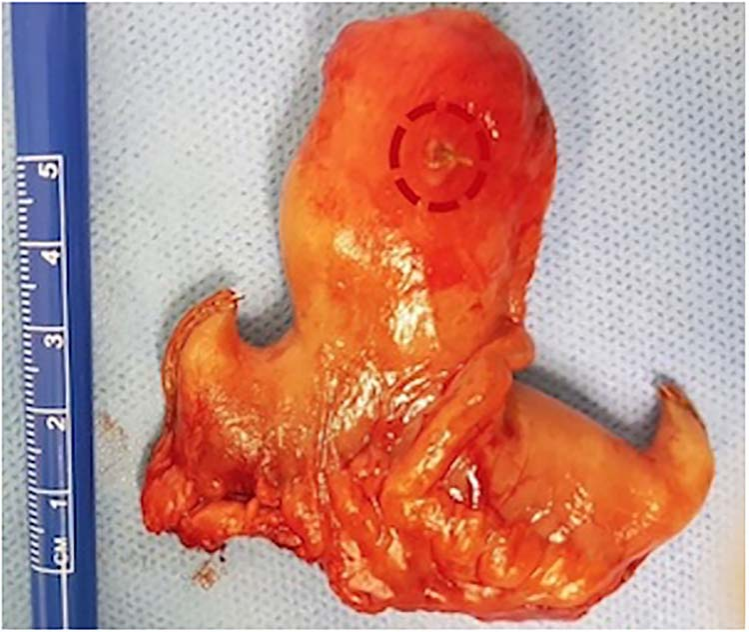
Postoperative specimen from small bowel segmental resection including the perforated by a fish bone Meckel’s diverticulum.

**Figure 4:**
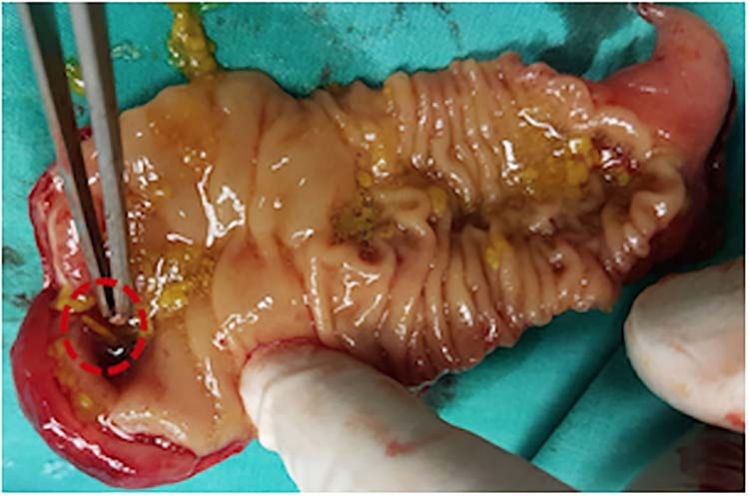
Postoperative specimen from small bowel segmental resection including the perforated by a fish bone Meckel’s diverticulum.

The patient underwent an emergent exploratory laparotomy. Intraoperatively, distended small bowel coils and a hypertrophic but non-inflammatory appendix vermiformis were discovered. During the small bowel examination, adhesions as well as a large about one and a half inches long, inflammatory, and perforated by a fishbone Meckel’s diverticulum was observed about 80 cm from the ileocecal valve (Fig. 3), solidly attached to the urinary bladder. Adhesiolysis, segmental small bowel resection incorporating the Meckel’s diverticulum, and a side-to-side small bowel anastomosis were carried out (Fig. 4). Urinary bladder leakage was discovered during the Douglas pouch examination, and the bladder wall deficit was then closed via a double-layer suture pattern. A drainage tube inserted and positioned in Douglas’s pouch to detect any early postoperative leakage. The patient’s postoperative course was uneventful and the patient discharged home on the sixth postoperative day, and the Foley catheter was removed on the tenth postoperative day.

## DISCUSSION

Meckel’s diverticulum is the most common congenital anomaly of gastrointestinal tract characterized by a blind pouch protruding from the wall of the small intestine [2, 4]. It is a rare condition, occurring in approximately 2% of the population [3]. While Meckel’s diverticulum is usually asymptomatic, it can cause various complications such as intussusception, intestinal obstruction, ulceration, bleeding, diverticulitis, perforation and, very rarely, neoplasms and vesicoenteric fistulas [1, 4].

Vesicoenteric fistulas (VEFs) are abnormal connections between the urinary bladder and the intestine that can result in the leakage of urine into the intestinal lumen. These fistulas are rare complications, with an incidence of 0.1%–0.2% in the general population [2]. Meckel’s diverticulum is a rare cause of vesicoenteric fistulas, accounting for less than 5% of all cases while most commonly occur secondary to diverticulitis, Crohn’s disease, colon and bladder malignancies [6]. Recent literature has documented eleven similar cases, with Table 2 offering a comprehensive summary encompassing their symptoms, the diagnostic imaging techniques employed, and the surgical interventions performed. The cause of the formation of vesicointestinal fistulas to the reported cases included the following: unknown etiology—idiopathic in seven cases [2, 3, 5, 7–10], Crohn disease [11], enterolith, adenocarcinoma of ectopic pancreatic tissue, and foreign body [12–14].

**Table 2:** Eleven similar studies have been documented reporting vesicoenteric fistulas formation between Meckel’s diverticulum and urinary bladder

Author	Sex	Age	Symptoms	Imaging tests	Treatment
Dearden [8]	Female	81	pain in iliac fossa, dysuria, frequency, nausea, vomiting	cystoscopy	Laparotomy
MacKenzie [9]	Female	30	anorexia, nausea, diarrhea, weakness	voiding cystourethrogram	Laparotomy
Petros [11]	Male	22	fever, dysuria	cystoscopy	Laparotomy
Hudson [12]	Male	23	abdominal pain, diarrhea	CT	Laparotomy
Graziotti [13]	Male	40	abdominal pain, dysuria, low urinary tract infections, hematuria	CT, MRI, cystoscopy	Laparotomy
Bouassida [2]	Female	66	abdominal pain with distension, fever, dysuria, nausea, vomiting	CT, cystoscopy	Laparotomy
Bourguida [3]	Male	35	abdominal pain with distension, absence of bowel movements	CT	Laparotomy
Fujita [14]	Male	58	dysuria, frequency	CT, MRI, cystoscopy	Laparotomy
Hakoda [7]	Male	46	hematuria	CT, MRI, cystoscopy	Laparoscopy
Murphy [10]	Male	44	lower abdominal pain	CT	Laparotomy
Han [5]	Male	51	chronic urinary tract infections, frequency	CT, cystoscopy	Laparoscopy

VEFs can be difficult to diagnose, as symptoms may be nonspecific and the condition is rare [5]. Symptoms of vesicoenteric fistulas can include abdominal pain, fever, lower urinary tract symptoms, urinary retention, and vomiting [6, 7]. The diagnosis of these conditions can be made through a combination of clinical examination, laboratory tests, and imaging studies, such as ultrasound, computed tomography (CT), magnetic resonance imaging (MRI), cystoscopy, Tc-99 m DTPA or gastrointestinal (GI) contrast studies [6]. In some cases, including ours, laparotomy may be necessary to confirm the diagnosis [11]. In our case, a vesicoenteric fistula could not be seen in the emergent CT examination of the abdomen. The diagnosis was confirmed intraoperatively with the existence of a vesicoenteric fistula developed between the perforated by a foreign body (fishbone) Meckel’s diverticulum and the urinary bladder.

The surgical approach for the treatment of vesicoenteric fistulas incorporating Meckel’s diverticulum typically involves the removal of the affected portion of the intestine and the Meckel’s diverticulum, as well as repair of the fistula [7]. In some cases, additional procedures such as appendectomy may be necessary [2]. After surgery, the patient may need to undergo urinary catheterization and may require ongoing monitoring for any complications [5]. Laparoscopic surgery can successfully be used to treat vesicoenteric fistula due to Meckel’s diverticulum [7]. Operative management of vesicoenteric fistulas involves resection and reanastomosis of the bowel segment causing the fistula and closing of the bladder [6].

## CONCLUSION

In conclusion, Meckel’s diverticulum is a rare cause of vesicoenteric fistula formation. It could lead to life-threatening complications due to foreign body ingestion or perforation. The diagnosis of vesicoenteric fistula poses a challenge even for an experienced radiologist. General surgeons should be aware of this scarcity because of the provocative treatment required. Treatment is mainly surgical and involves the removal of the affected portion of the intestine incorporating the Meckel’s diverticulum, as well as repair of the fistula. Further studies should be conducted to evaluate the safety and effectiveness of laparoscopic surgery for the treatment of vesicoenteric fistulas incorporated Meckel’s diverticulum.
